# Supplementary material for: Engineered Lactococcus lactis secreting Flt3L and OX40 ligand for in situ vaccination-based cancer immunotherapy
Source: Nat Commun. 2022 Dec 3;13:7466. doi: 10.1038/s41467-022-35130-7 (PMC9719518; doi:10.1038/s41467-022-35130-7)
Supplement: Supplementary file 1 — Supplementary Information [file 41467_2022_35130_MOESM1_ESM.pdf]

Supplementary Information

Engineered *Lactococcus lactis* secreting Flt3L and OX40 ligand for in situ vaccination-based cancer immunotherapy

Junmeng Zhu[1†], Yaohua Ke[1†], Qin Liu[1†], Ju Yang[1], Fangcen Liu[2], Ruihan Xu[1], Hang Zhou[1], Aoxing Chen[1], Jie Xiao[1], Fanyan Meng[1], Lixia Yu[1], Rutian Li[1], Jia Wei[1] and Baorui Liu[1✉]

[1]The Comprehensive Cancer Centre of Nanjing Drum Tower Hospital. The Affiliated Hospital of Nanjing University Medical School. 321 Zhongshan Road, Nanjing 210008, China.

[2]Department of Pathology. The Affiliated Hospital of Nanjing University Medical School. 321 Zhongshan Road, Nanjing 210008, China.

[†]These authors contributed equally: Junmeng Zhu, Yaohua Ke, Qin Liu.

[✉]email: baoruiliu@nju.edu.cn

a

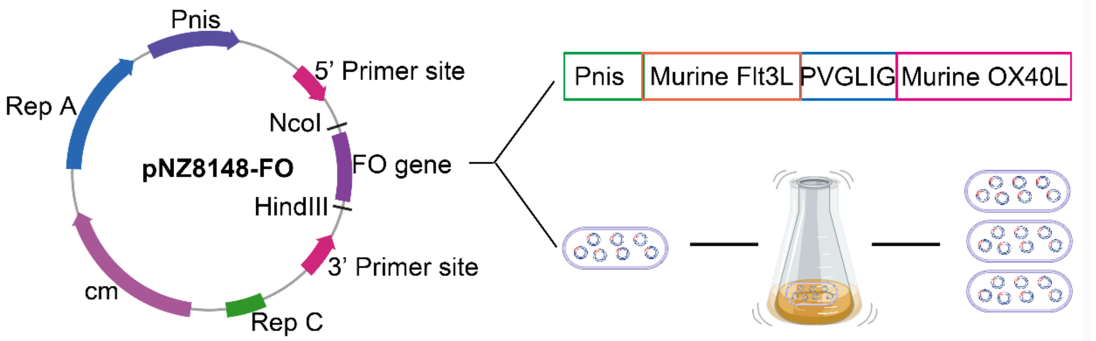

b

MHHHHHHPVGLIGGTPDCYFSHSPISSNFKVKFRELTDHLLKDYPVTVAVNLQDEKHCKALW
SLFLAQRWIEQLKTVAGSKMQTLLEDVNTEIHFVTSCTFQPLPECLRFVQTNISHLLKDTCTQL
LALKPCIGKACQNFSR**CLEVQCQPDSSTLLPPR**SPIALEATELPEPRPRQPVGLIGSSSPAKD
PPIQRLRGAVTR**CEDGQLFISSYKNEYQTMEVQNNSVVIK**CDGLYIIYLKGSFFQEVKIDLHF
REDHNPISIPMLNDGRRIVFTVVASLAFKDKVYLTVNAPDTLCEHLQINDGELIVVQLTPGYCAP
EGSYHSTVNQVPL

c

Protein name	Accession	Theoretical PI	Theoretical MW(KDa)	Mascot Score/No. of Match Peptides	Protein Coverage(%)
**Flt3L**	P49772	8.35	26.1	3179/15	66
**OX40L**	P43488	6.52	22.2	1966/10	37

Supplementary Figure 1, Mass spectrographic analysis of the Flt3L-OX40L fusion protein expressed by FOLactis. a, Plasmid map of a *Lactococcus lactis-Escherichia coli* shuttle vector using pNZ8148 expressing the Flt3L-OX40L fusion protein (FO). Created with BioRender.com. **b**, The amino acid sequence of FO. Flt3L sequence in orange, PVGLIG sequence in blue and OX40L sequence in pink. Two of the best matched peptides were labeled in red. **c**, The matched protein information of the mass spectrometric analysis.

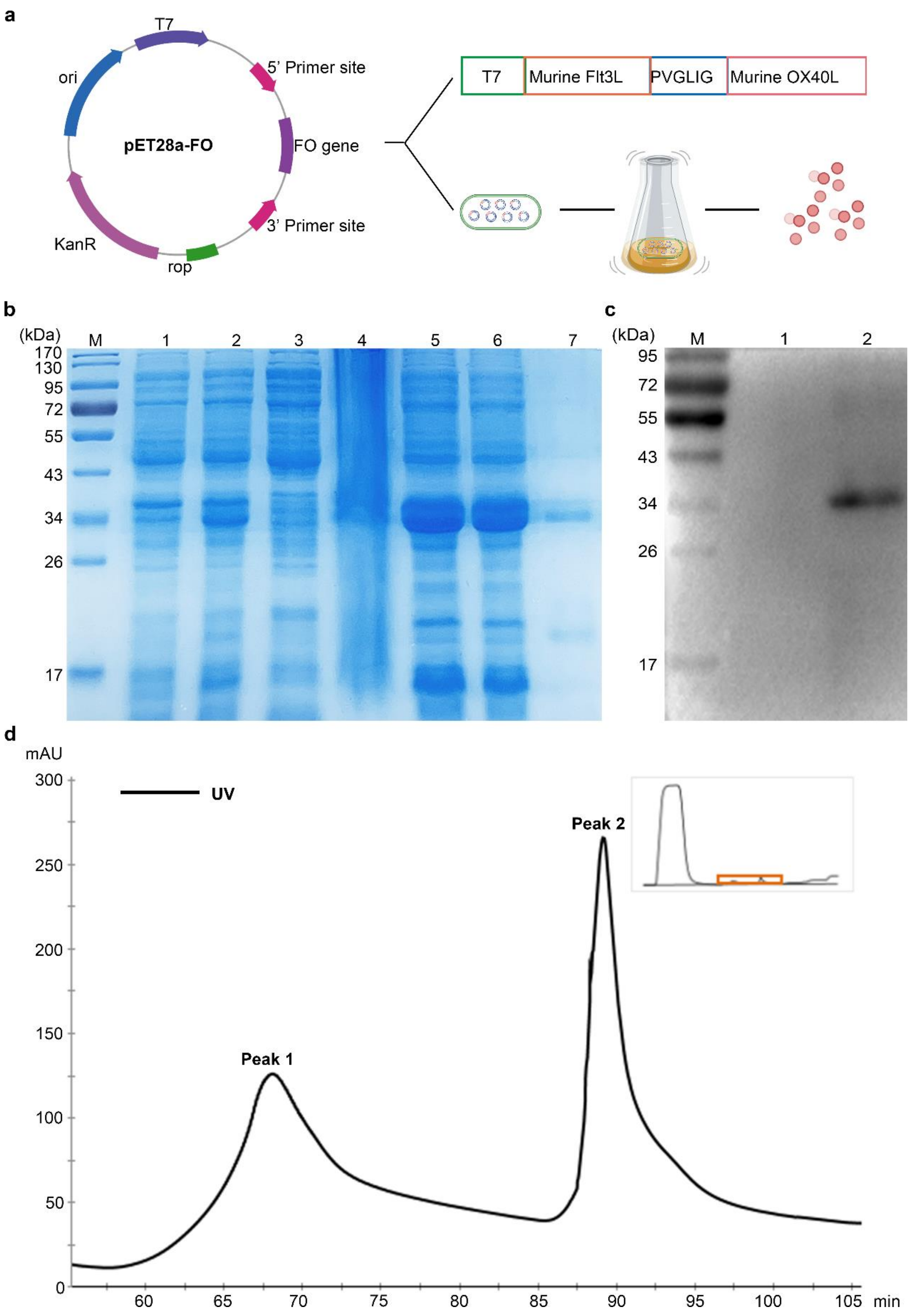

Supplementary Figure 2, Expression and purification analysis of recombinant protein Flt3L-OX40L. **a**, Plasmid map of pET28a expressing the Flt3L-OX40L fusion protein (FO). Created with BioRender.com. **b**, SDS-PAGE of the Flt3L-OX40L fusion protein (FO) (One representative data was shown from 3 independently repeated experiments). Lane M, protein

ladder; Lane 1, bacteria without isopropyl β-D-1-thiogalactopyranoside (IPTG) induction; Lane 2, bacteria induced with 1 mM IPTG; Lane 3, the supernatant of the ultrasonic lysate; Lane 4, the precipitation of the ultrasonic lysate; Lane 5, solubilized inclusion bodies; Lane 6, refolded products; Lane 7, purified and refolded products. IPTG: the protein expression inducer. **c**, Eluted fractions were identified by western blot analysis (One representative data was shown from 3 independently repeated experiments). Lane M, protein ladder; Lane 1, bacteria without IPTG induction; Lane 2, purified FO. **d**, Purification profile of refolded FO. Peak1, impure protein; Peak 2, purified FO. UV, ultraviolet; AU: absorbance unit; min: minutes.

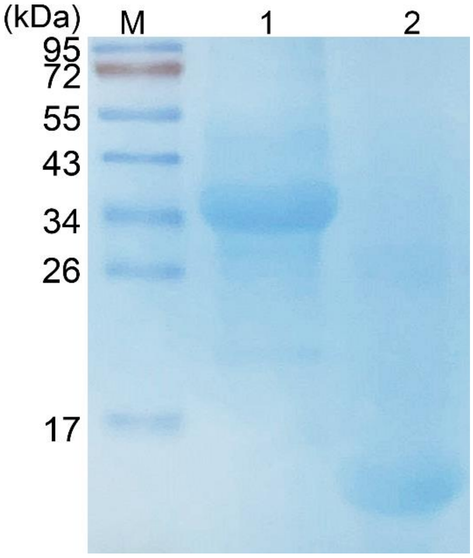

Supplementary Figure 3, MMP2/9 sensitive peptide PVGLIG could be cut by MMP2/9. We used collagenase to verify whether Flt3L-OX40L fusion protein (FO) could be cut by MMP2/9. Purified FO was extracted from lysates of FOLactis using metal affinity chromatography by the ÄKTA fast protein liquid chromatography system according to the manufacturer's instructions. After cleaving by collagenase (including MMP2/9, 1 mg ml^{-1}) for 5 hours, most FO could be divided, as shown in the SDS-PAGE (One representative data was shown from 3 independently repeated experiments). The mass ratio of FO to collagenase is 2:1. Lane M, protein ladder; Lane 1, Purified FO extracted from lysates of FOLactis; Lane 2, Purified FO cleaved by collagenase (including MMP2/9, 1 mg ml^{-1}).

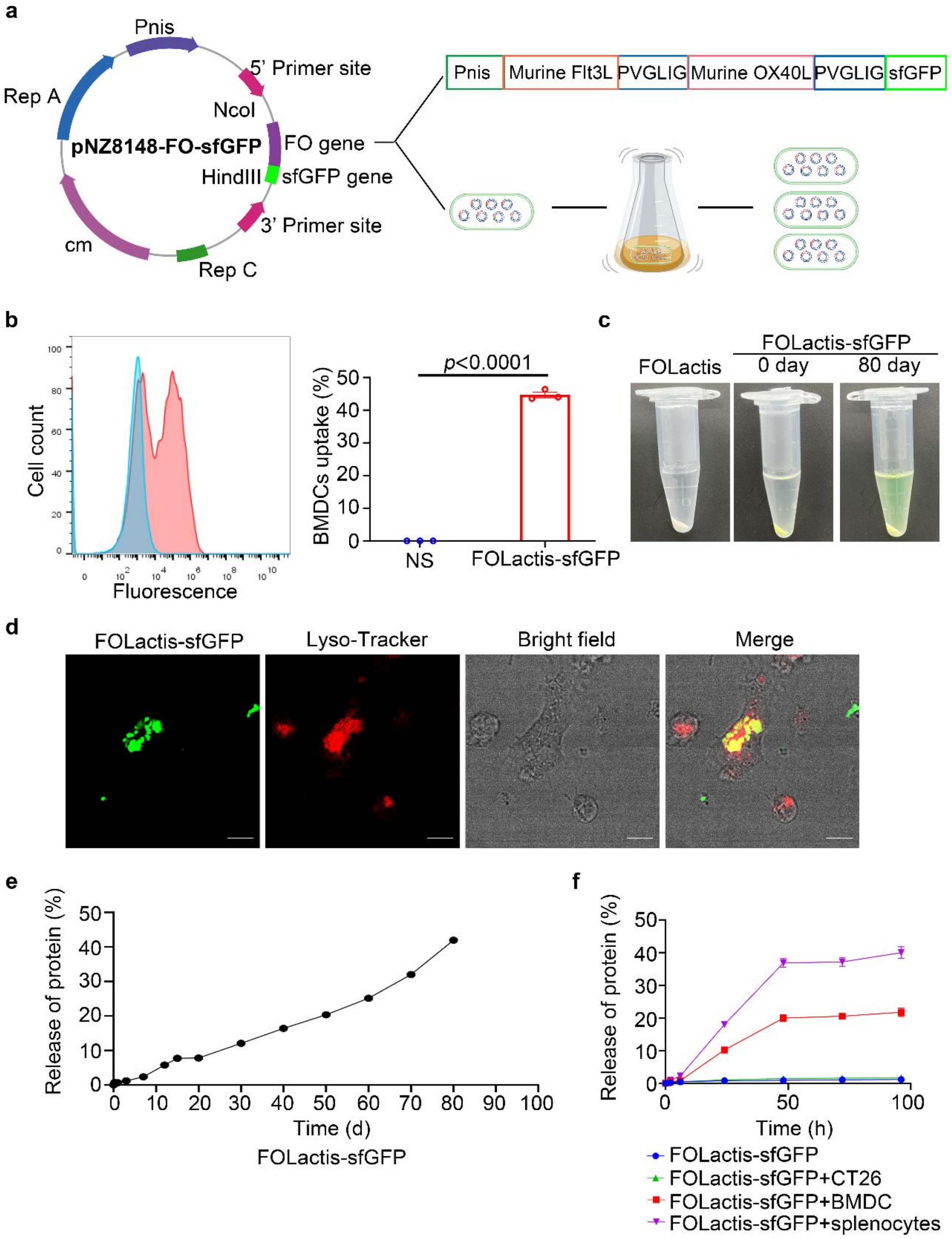

Supplementary Figure 4, Sustained release of the fusion protein from the engineered Lactis. a, Plasmid map of a *Lactococcus lactis-Escherichia coli* shuttle vector using pNZ8148 expressing the Flt3L-OX40L-sfGFP fusion protein. Created with BioRender.com. **b**, Cellular uptake of FOLactis-sfGFP after a two-hour incubation with BMDCs, as assessed by flow cytometry (n=3, biologically independent samples). The error bars represented mean ± s.e.m. Statistical significance was determined by two-tailed unpaired Student's t-tests. ns represented

$p > 0.05$. **c**, 10^9 CFU FOLactis or FOLactis-sfGFP were resuspended in PBS at 37 °C. In vitro images to demonstrate the activity and release of the Flt3L-OX40L-sfGFP fusion protein. **d**, Colocalization analysis of FOLactis-sfGFP (green) with lysosomal compartments (red) in BMDCs by confocal microscopy (two-hour incubation) (One representative data was shown from 3 independently repeated experiments). White scale bars, 10 μm. **e-f**, Curves of the Flt3L-OX40L-sfGFP fusion protein release from the engineered Lactis with or without other cells in PBS at 37 °C (n=5). The error bars represented mean ± s.e.m. Source data are provided as a Source Data file.

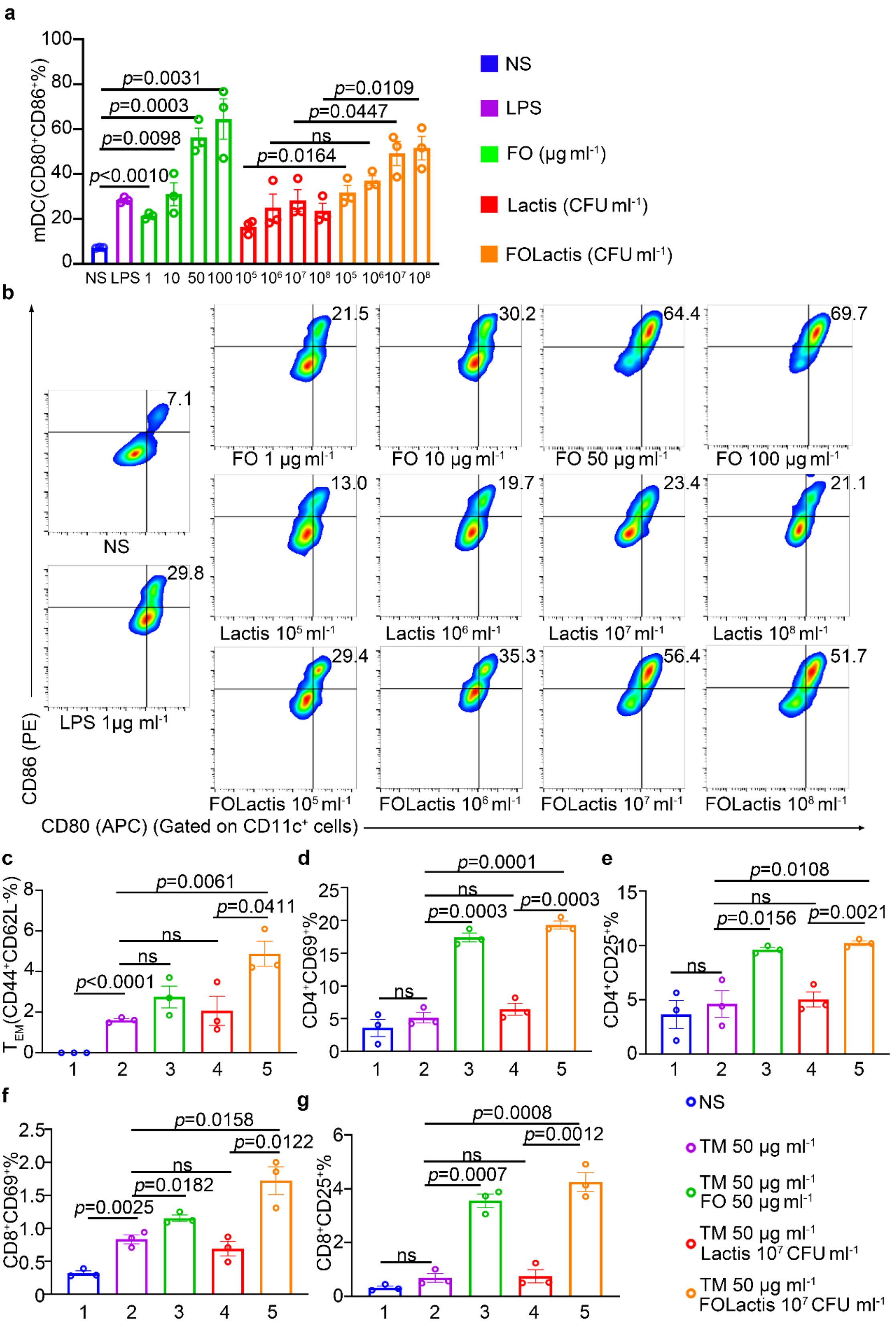

Supplementary Figure 5, The Flt3L-OX40L fusion protein (FO) and FOLactis efficiently induced the activation of DCs and T cells in vitro. a, The percentage of mature DCs (mDCs, $CD11c^+CD80^+CD86^+$) after co-incubation with different concentrations of FO or the crude

lysates from Lactis or the crude lysates from FOLactis in vitro for 24 hours (n=3). **b**, Representative flow cytometry images of mDCs after co-incubation with different concentrations of FO or the crude lysates from Lactis or the crude lysates from FOLactis in vitro for 24 hours. **c**, The percentage of the effector memory T cells (T_{EM}, $CD3^+CD8^+CD44^+CD62L^-$) in the T cells from the spleen of BALB/c mouse stimulated by 50 μg ml^{-1} TM (without or with FO, the crude lysates from Lactis, the crude lysates from FOLactis) for 10 days in vitro (n=3). TM, autologous tumour cell membranes. **d-g**, The quantification of CD69 and CD25 expression on $CD8^+$ and $CD4^+$ T-cell subsets in the T cells from the spleen of BALB/c mouse stimulated by 50 μg ml^{-1} TM (without or with FO, the crude lysates from Lactis, the crude lysates from FOLactis) for 10 days in vitro (n=3). For **a**, **c-g**, data were mean ± s.e.m. statistical significance was determined by two-tailed unpaired Student's t-tests. ns represented $p>0.05$. Source data are provided as a Source Data file.

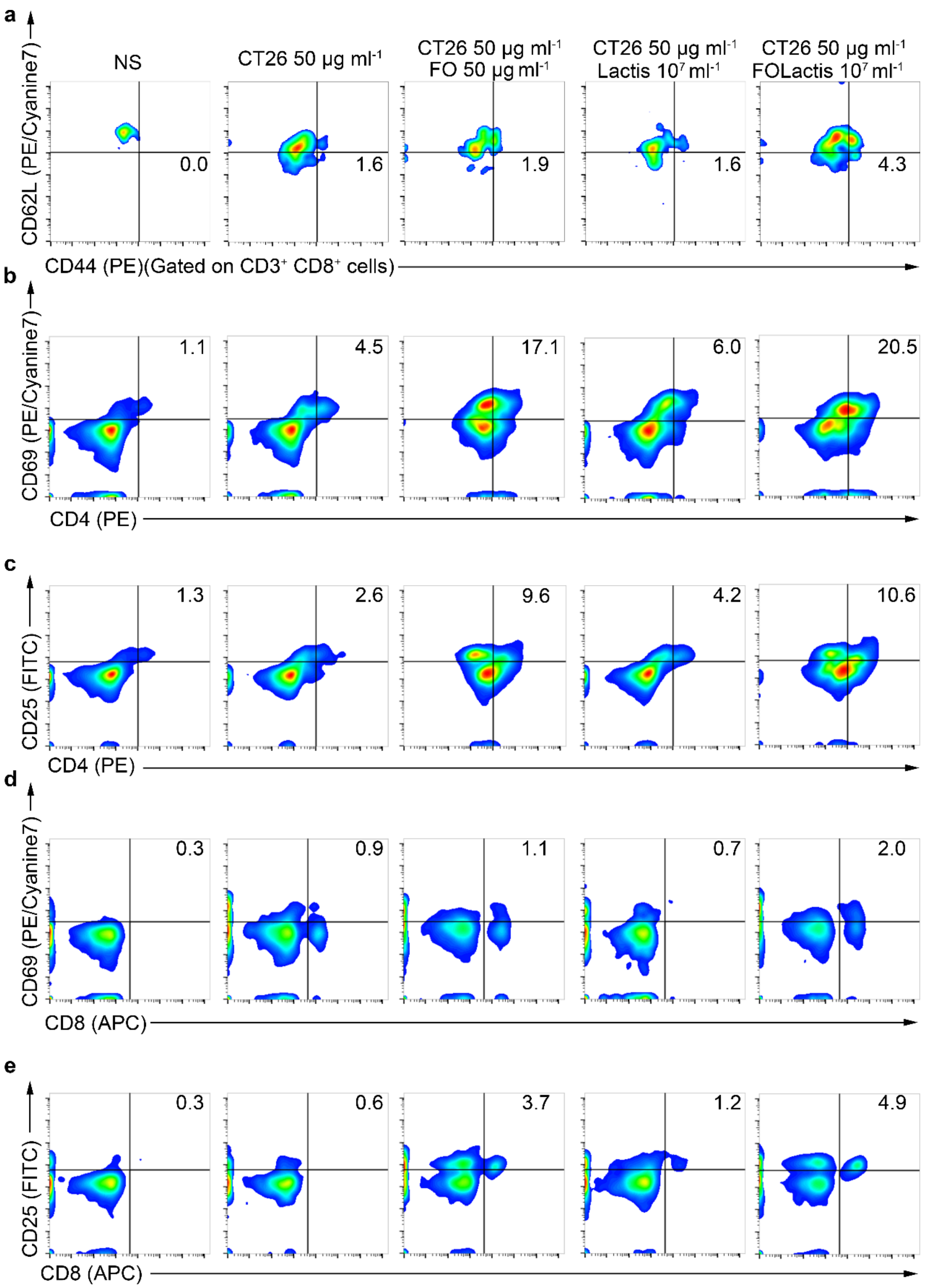

Supplementary Figure 6, The Flt3L-OX40L fusion protein (FO) and FOLactis efficiently induced T cell activation in vitro. a, Representative flow cytometry images of T_{EM} in the T cells from the spleen of BALB/c mouse stimulated by 50 μg ml^{-1} autologous tumour cell membranes (without or with FO, the crude lysates from Lactis, the crude lysates from FOLactis)

for 10 days in vitro. **b-e**, Representative flow cytometry images of CD69 and CD25 expression on CD8⁺ and CD4⁺ T-cell subsets in the T cells from the spleen of BALB/c mouse stimulated by 50 μg ml^{-1} autologous tumour cell membranes (without or with FO, the crude lysates from Lactis, the crude lysates from FOLactis) for 10 days in vitro.

a

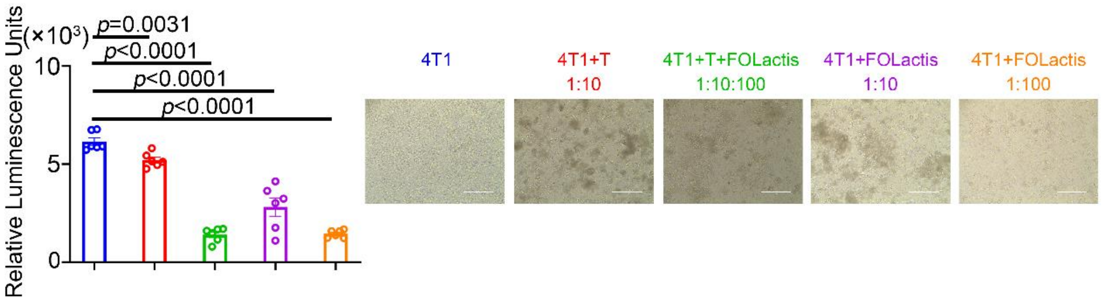

b

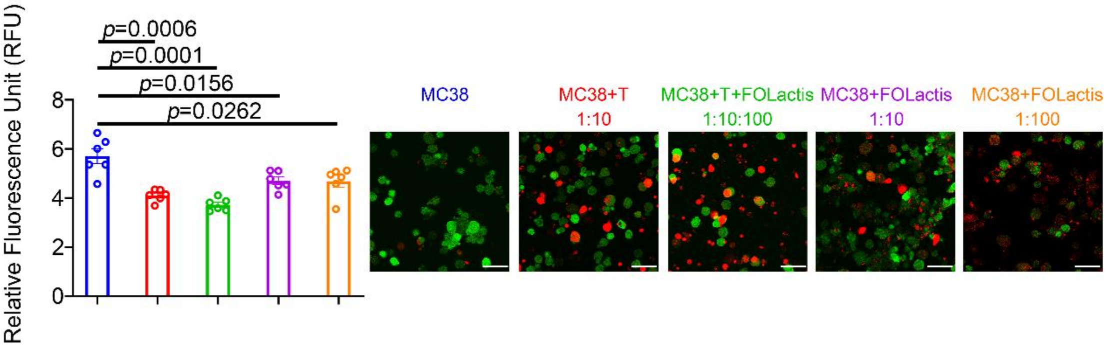

Supplementary Figure 7, FOlactis could kill cancer cells by direct cell lysis. a, Luminescence was detected by the addition of D-luciferin to 4T1-GFP-Luc cells after incubated with T cells and/or FOLactis for 24 hours (n=6). Cells were examined under the microscope EVOS FL Auto Cell Imaging System (Invitrogen) and photographed. White scale bars, 200 μm. **b**, Fluorescence was detected after MC38-GFP-Luc cells incubated with T cells and/or FOLactis for 24 hours (n=6). Cells were examined under a confocal laser scanning microscopy (Leica, Germany). White scale bars, 50 μm. Data were mean ± s.e.m. statistical significance was determined by two-tailed unpaired Student’s t-tests. ns represented p>0.05. Source data are provided as a Source Data file.

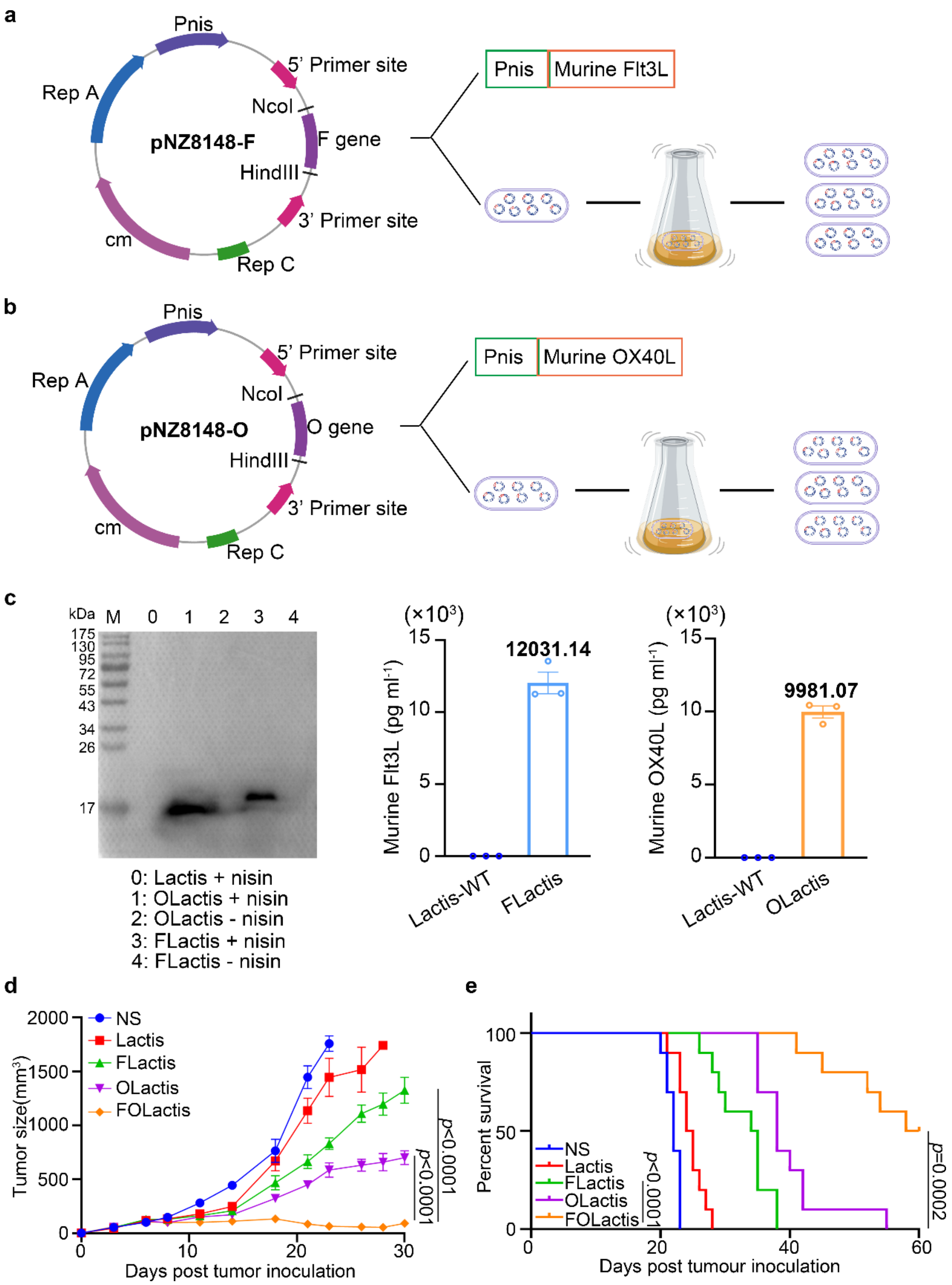

Supplementary Figure 8, Construction of engineered *Lactococcus lactis* delivering Flt3L (FLactis) or OX40L (OLactis). a-b, Plasmid map of a *Lactococcus lactis-Escherichia coli* shuttle vector using pNZ8148 expressing Flt3L (FLactis) or OX40L (OLactis). Created with BioRender.com. **c**, Western blotting analysis of the induced or non-induced engineered *Lactococcus lactis*. Nisin is an inducer of protein expression. M: molecular mass marker; Lane 0-4: the whole bacteria lysates (bacteria) of wild-type *Lactococcus lactis* (Lactis) induced by

nisin, OLactis induced by nisin, non-induced OLactis, FLactis induced by nisin and non-induced FLactis, respectively. Bacteria (10^9 CFU) were collected and the pellets were sonicated. The amounts of the target protein in the bacterial lysates of FLactis / OLactis were assessed by ELISA (One representative data was shown from 3 independently repeated experiments). **d**, Average tumour-growth curves of BALB/c mice bearing CT26 colon tumour with different treatments as indicated (n=6). The mice were administered with NS, 10^9 CFU Lactis, 10^9 CFU FLactis, 10^9 CFU OLactis or 10^9 CFU FOLactis intratumourally on days 7, 9 and 11, which were dissolved in normal saline to a final volume of 100 μl per dose. The tumour size was measured every 2-3 days from the first administration day. The error bars represented mean ± s.e.m. *p*-values were calculated by two-way ANOVA and Tukey post-test and correction. ns represented $p>0.05$. **e**, Survival curves of BALB/c mice in different groups for 60 days (n=6). *p*-values were calculated by log-rank (Mantel-Cox) test. ns represented $p>0.05$. Source data are provided as a Source Data file.

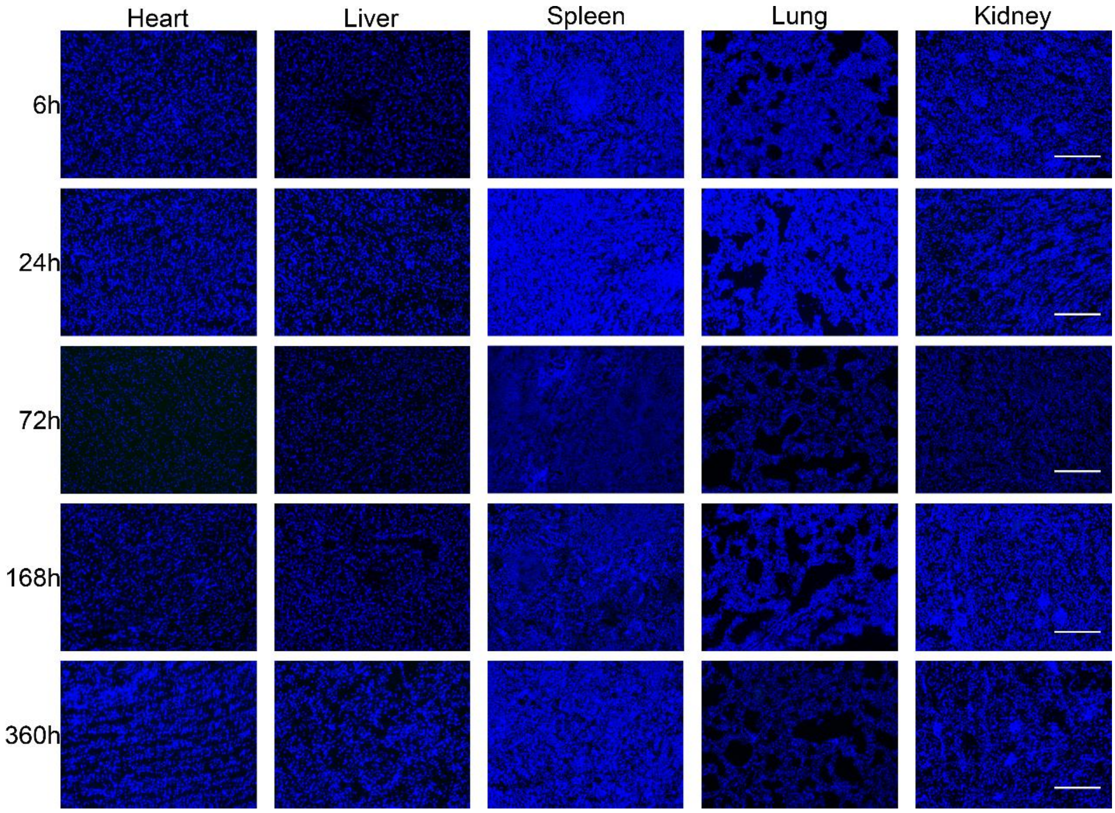

Supplementary Figure 9, Biodistribution of FOLactis. Frozen sections of hearts, livers, spleens, lungs, and kidneys in CT26 mouse colon tumour model at 6, 24, 72, 168, 360 hours after intratumoural injection of 10^9 CFU FOLactis (n=3). FOLactis were labeled with DiO (green);

nucleus, blue. White scale bars, 200 μm.

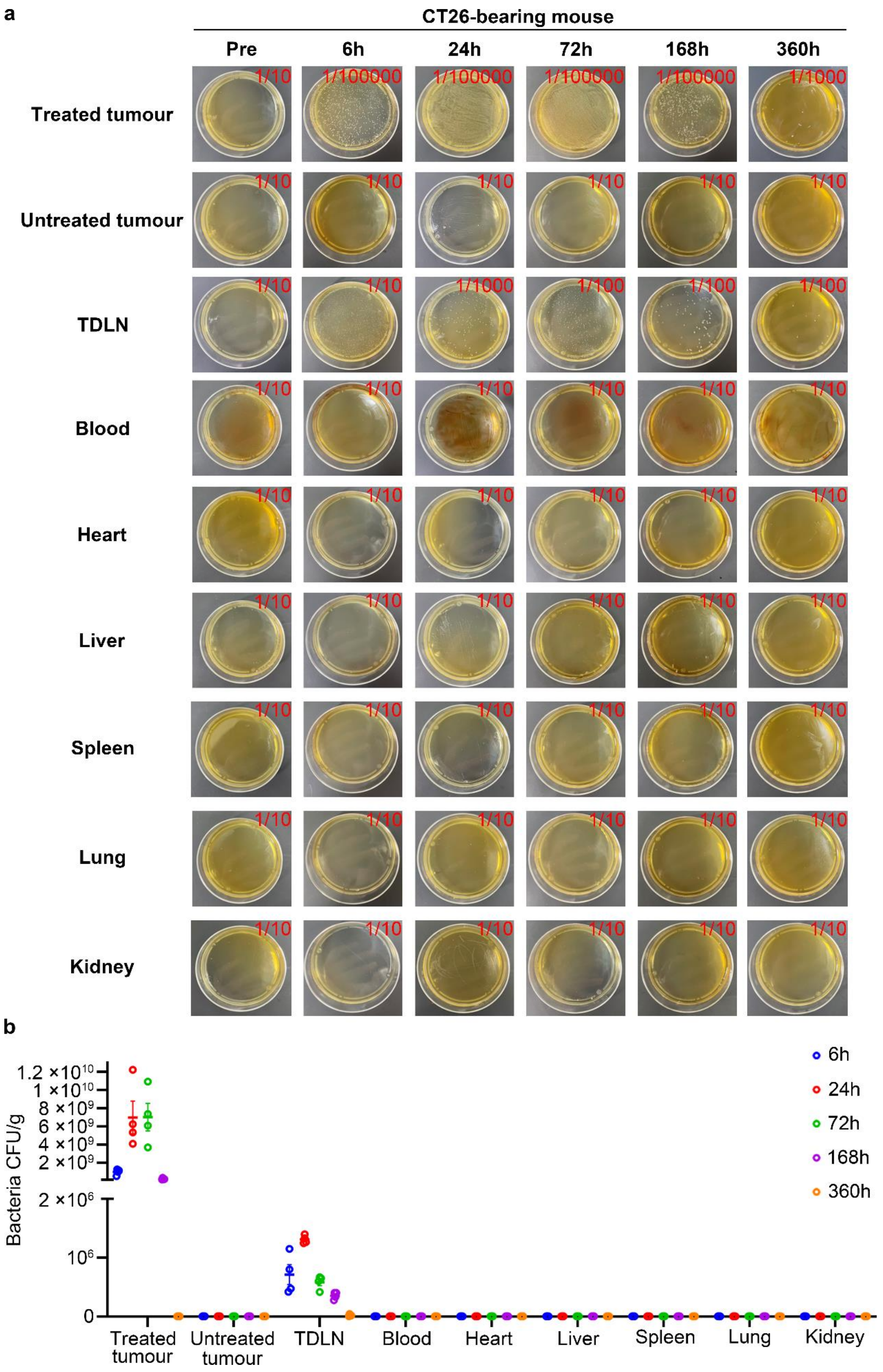

Supplementary Figure 10, Bacterial colonization in CT26-bearing mice after intratumoural injection. a-b, Representative photographs of solid GM17 agar plates (**a**) and quantification (**b**) of bacterial colonization in various organs harvested from CT26-bearing mice at different time points after injection of bacteria (n=4, biologically independent samples). The error bars represented mean ± s.e.m. Source data are provided as a Source Data file.

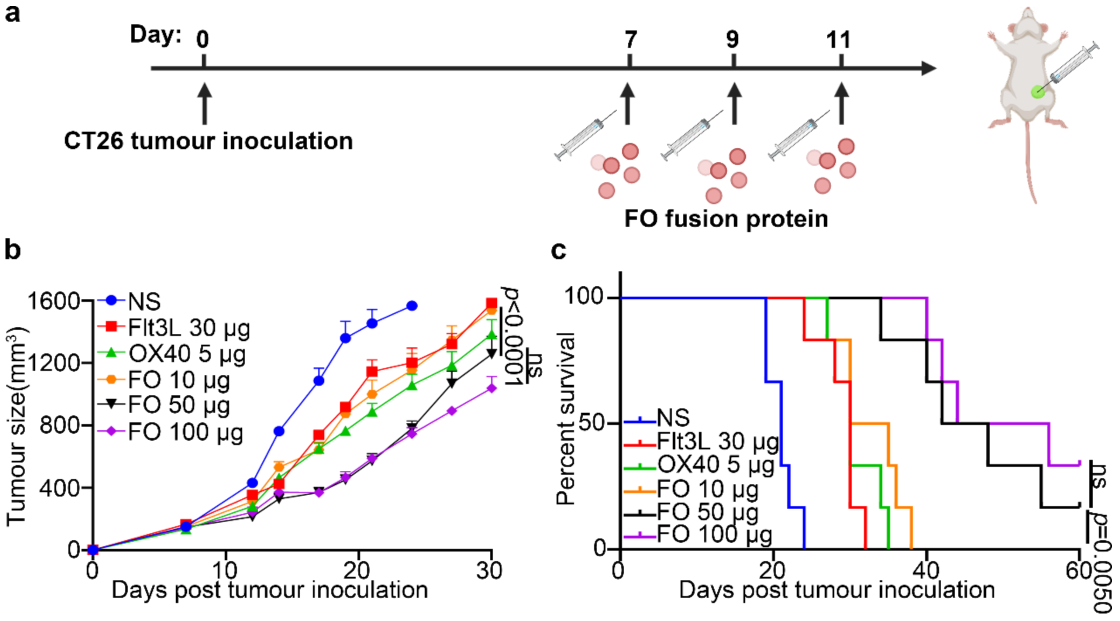

Supplementary Figure 11, In vivo antitumour effect of the Flt3L-OX40L fusion protein (FO). a, Schematic diagram of the therapeutic treatment in male CT26 tumour-bearing mice. BALB/c mice were implanted with CT26 cells (5×10^5) on the left lower sides of the abdomen on day 0, and received treatments on days 7, 9 and 11. Created with BioRender.com. **b,** Average tumour-growth curves of BALB/c mice bearing CT26 colon tumour with different treatments as indicated (n=5). The mice were administered with NS, 30 μg Flt3L, 5 μg OX40, 10 μg FO, 50 μg FO, or 100 μg FO intratumourally on days 7, 9, and 11, which were dissolved in normal saline to a final volume of 100 μl per dose. The tumour size was measured every 2-3 days from the first administration day. The error bars represented mean ± s.e.m. *p*-values were calculated by two-way ANOVA and Tukey post-test and correction. ns represented $p>0.05$. **c,** Survival curves of BALB/c mice in different groups for 60 days (n=6). *p*-values were calculated by log-rank (Mantel–Cox) test, ns represented $p>0.05$. Source data are provided as a Source Data file.

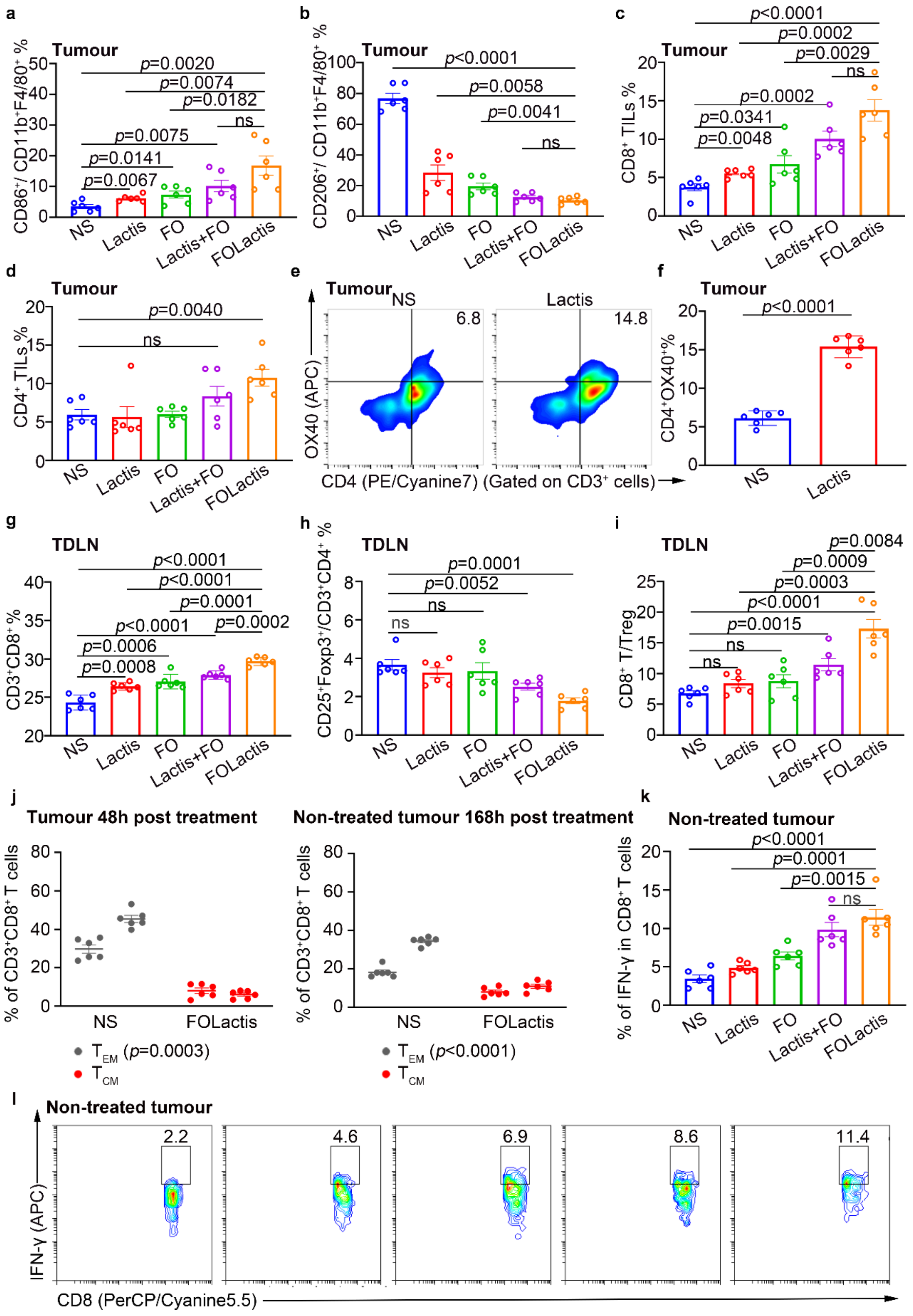

Supplementary Figure 12, In situ vaccination with FOLactis modulated the immune microenvironment in the local tumour and TDLN. a-d, CT26 tumour-bearing mice in different groups were sacrificed two days after the last treatment, and the proportions of immune cells were analyzed by flow cytometry of the tumours and TDLN (n=6). Shown are flow

cytometric analysis of M1-like macrophages (**a**, gate: CD11b$^+$F4/80$^+$ macrophages), M2-like macrophages (**b**, gate: CD11b$^+$F4/80$^+$ macrophages), CD3$^+$CD8$^+$ T cells (**h**, gate: lymphocytes) and CD4$^+$ T cells (**i**, gate: lymphocytes) in the TME. **e-f**, When intratumoural injection either with normal saline (NS) or Lactis once, tumours were excised 48 hours later, and OX40 expression of the CD3$^+$CD4$^+$ T cell subset was analyzed by flow cytometry (n=6). **g-h**, Flow cytometric analysis of CD3$^+$CD8$^+$ T cells (**g**, gate: lymphocytes) and CD25$^+$Foxp3$^+$Treg cells (**h**, gate: CD3$^+$CD4$^+$ T cells) (n=6). **i,** The ratios of CD8$^+$ to Treg (n=6). **j**, Flow cytometric analysis of effector memory T cells (T_{EM}, CD3$^+$CD8$^+$CD44$^+$CD62L$^-$) and central memory T cells (T_{CM}, CD3$^+$CD8$^+$CD44$^+$CD62L$^+$) in the treated tumour (48h after treatment) and non-treated tumour (168h after treatment) (n=6). **k-l**, Frequency of tumour infiltrating IFN-γ$^+$ within CD8$^+$ T cells (168h after treatment) (n=6). For the experiments in **a-d**, **f-k**, data were mean ± s.e.m. statistical significance was determined by analysis of two-tailed unpaired Student's t-tests. ns represented *p*>0.05. Source data are provided as a Source Data file.

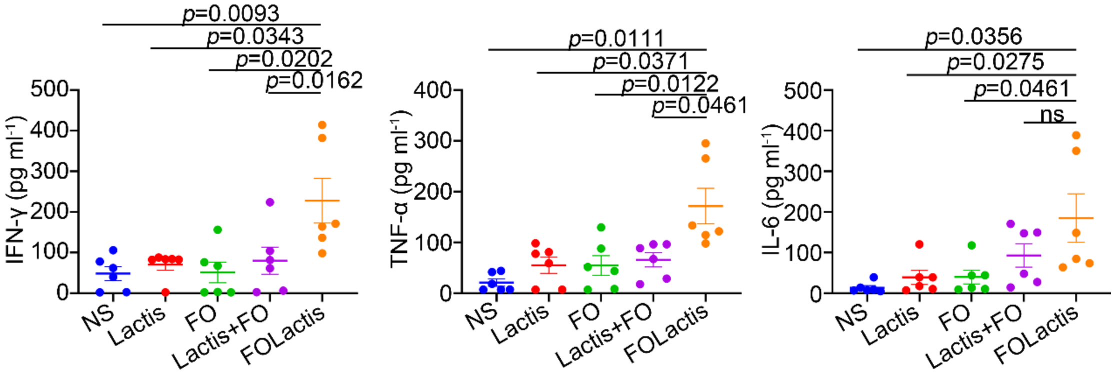

Supplementary Figure 13, In situ vaccination with FOLactis affected the levels of several cytokines. The interferon-γ (IFN-γ) level (left), TNF-α level (middle), and IL-6 level (right) in treated tumours from CT26 tumour-bearing mice isolated two days after the last treatment (n=6). Data were mean ± s.e.m. statistical significance was determined by analysis of two-tailed unpaired Student's t-tests. ns represented *p*>0.05. Source data are provided as a Source Data file.

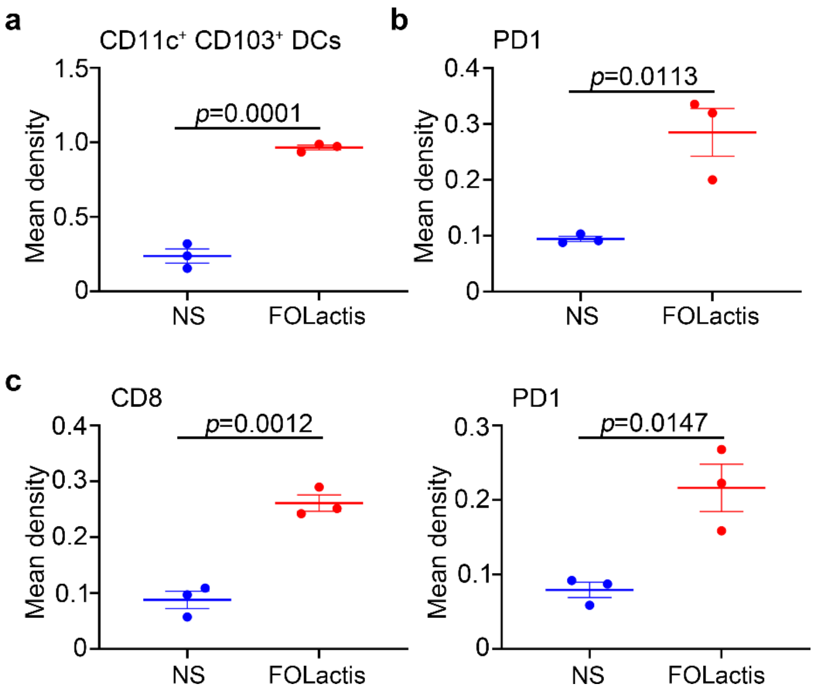

Supplementary Figure 14, Immunohistochemistry assay of the expression levels of $CD11c^{+}$ $CD103^{+}$ DCs, PD1, and CD8 in treated tumours. a-b, Histogram showing the expression levels of $CD11c^{+}$ $CD103^{+}$ DCs **(a)**, PD1 **(b)** in the NS group or the FOLactis group in CT26 tumour-bearing mice (n=3). **c**, Histogram showing the expression levels of CD8 and PD1 in the NS group or the FOLactis group in 4T1 tumour-bearing mice (n=3). Data were mean ± s.e.m. statistical significance was determined by analysis of two-tailed unpaired Student's t-tests. ns represented p>0.05. Source data are provided as a Source Data file.

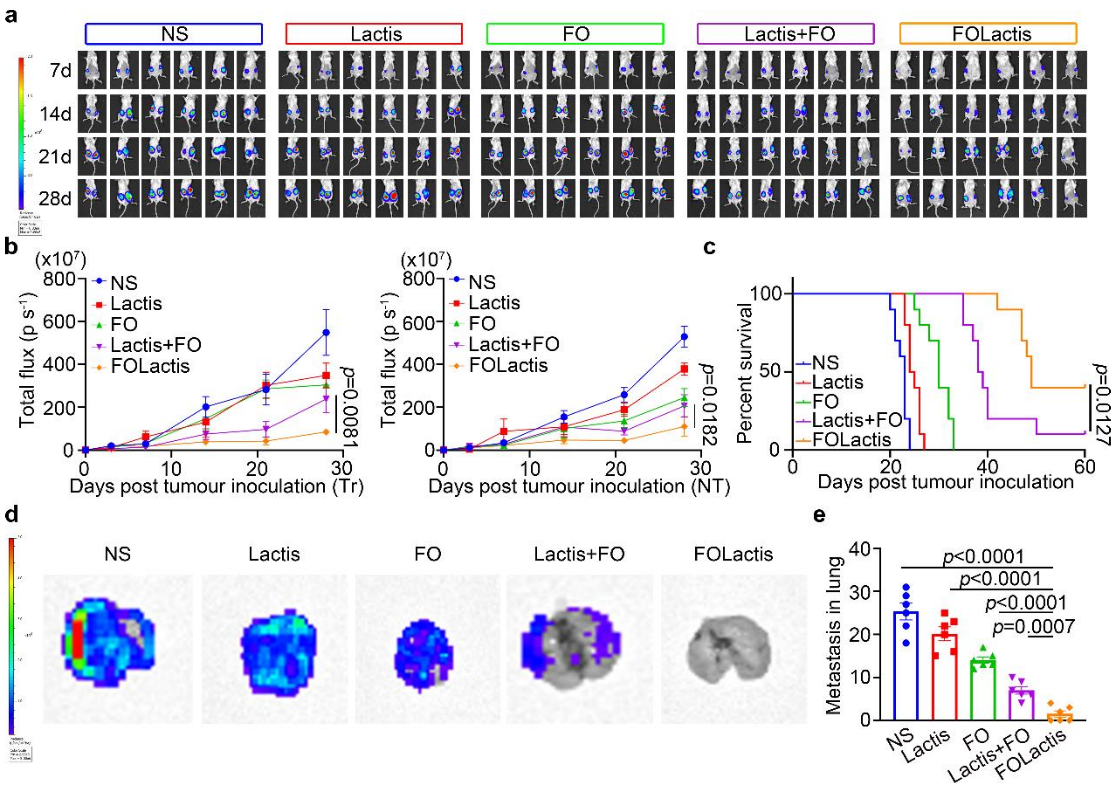

Supplementary Figure 15, FOLactis inhibited tumour growth and metastasis in the orthotopic 4T1 tumours. a-c, Orthotopic 4T1-bearing mice received treatments on days 7, 9, and 11 (n=6). Anterior bioluminescence images of tumour burden on days 7, 14, 21, and 28 after tumour inoculation **(a)**. Shown are the tumour signal **(b)** and overall survival **(c)**. **d**, Representative IVIS images of lungs extracted from 4T1-GFP-Luc orthotopic tumours. **e**, Quantification of metastatic lesions in lung tissues (n=6, biologically independent samples). For the experiments in b and e, data were the mean ± s.e.m. *p*-values were determined by two-tailed unpaired Student's t-tests. ns represented *p*>0.05. For the experiments in c, *p*-values were calculated by log-rank (Mantel-Cox) test. ns represented *p*>0.05. Source data are provided as a Source Data file.

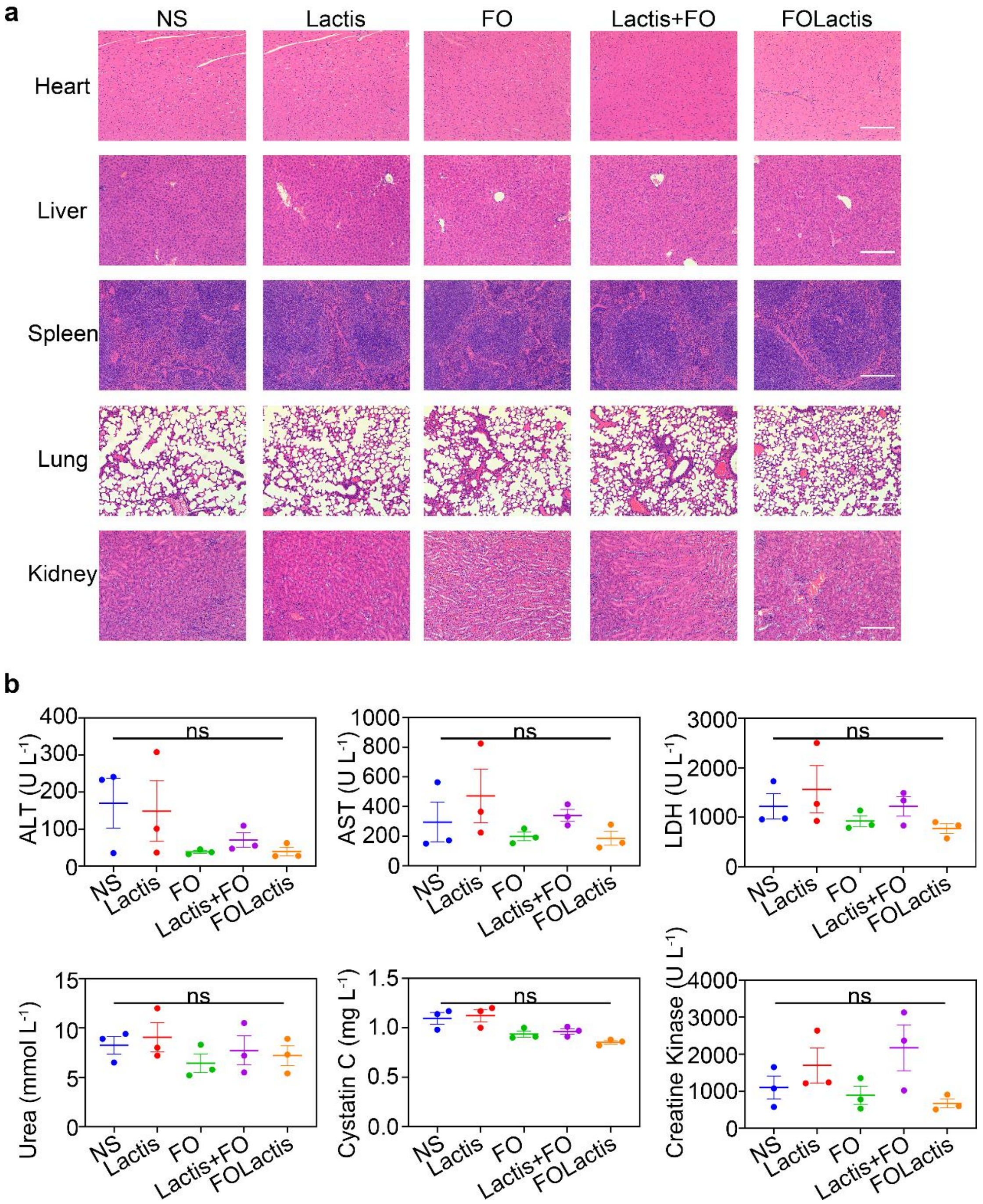

Supplementary Figure 16, Biosafety assessment of in situ vaccination with FOLactis. a, Hematoxylin-eosin staining of heart, liver, spleen, lung, and kidney in CT26 mouse tumour model seven days after the last treatment (n=3). The scale bars were 200 μm. **b,** Blood biochemistry and hematology data of male BALB/c mice seven days after the last treatment (n=3). ALT, glutamic-pyruvic transaminase; AST, aspartate aminotransferase; LDH, lactate dehydrogenase; Statistic was based on three mice per data point. Data were mean ± s.e.m. *p*-values were determined by two-tailed unpaired Student's t-tests. ns represented *p*>0.05. Source data are provided as a Source Data file.

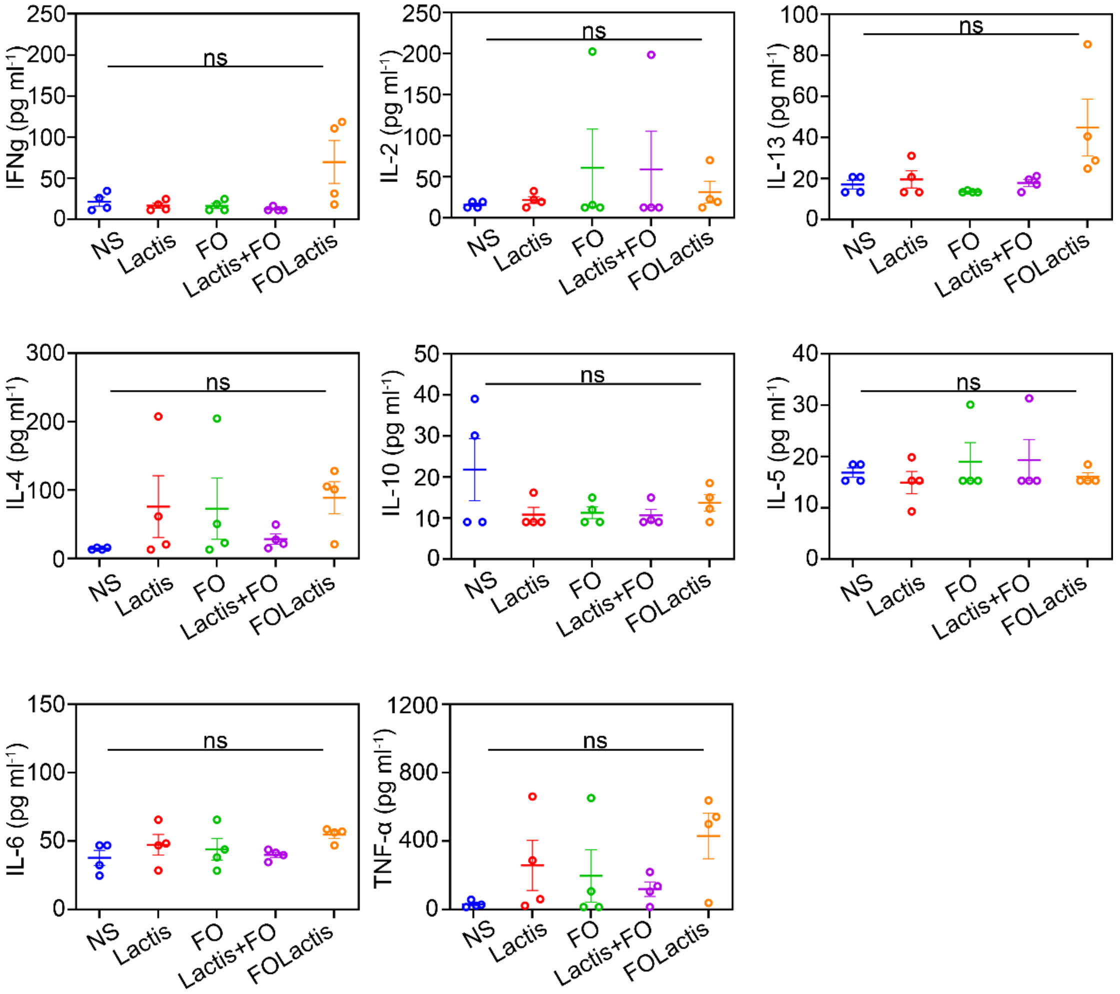

Supplementary Figure 17, Biosafety assessment of in situ vaccination with FOLactis. The levels of interferon-gamma (IFNg), IL-2, IL-13, IL-4, IL-10, IL-5, IL-6, and TNF-α in serum from CT26 tumour-bearing mice isolated seven days after the last treatment (n=4). Data were mean ± s.e.m. *p*-values were determined by two-tailed unpaired Student's t-tests. ns represented *p*>0.05. Source data are provided as a Source Data file.

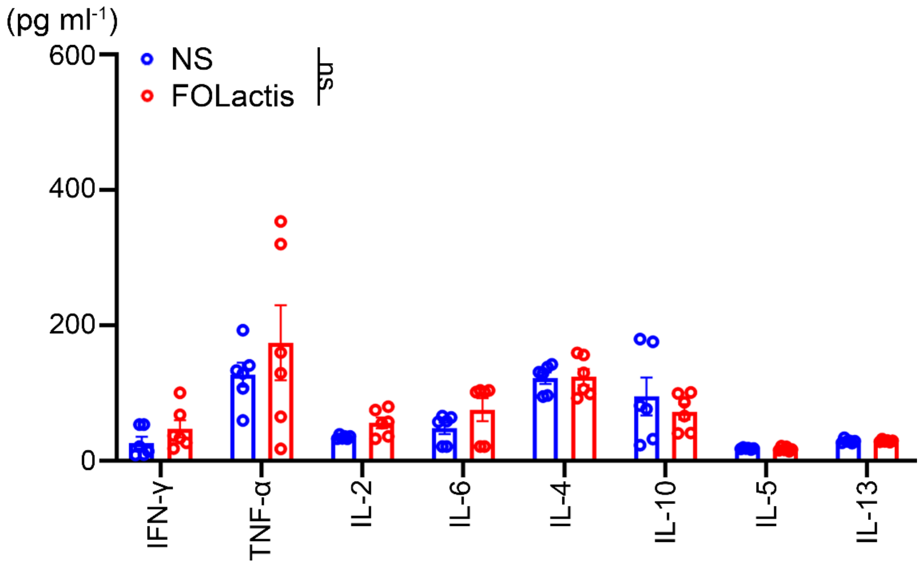

Supplementary Figure 18, FOLactis induced low systemic inflammation. The levels of interferon-gamma (IFNg), IL-2, IL-13, IL-4, IL-10, IL-5, IL-6, and TNF-α in serum from CT26 tumour-bearing mice isolated two days after the last treatment (n=6). Data were mean ± s.e.m. *p*-values were determined by two-tailed unpaired Student's t-tests. ns represented $p>0.05$. Source data are provided as a Source Data file.

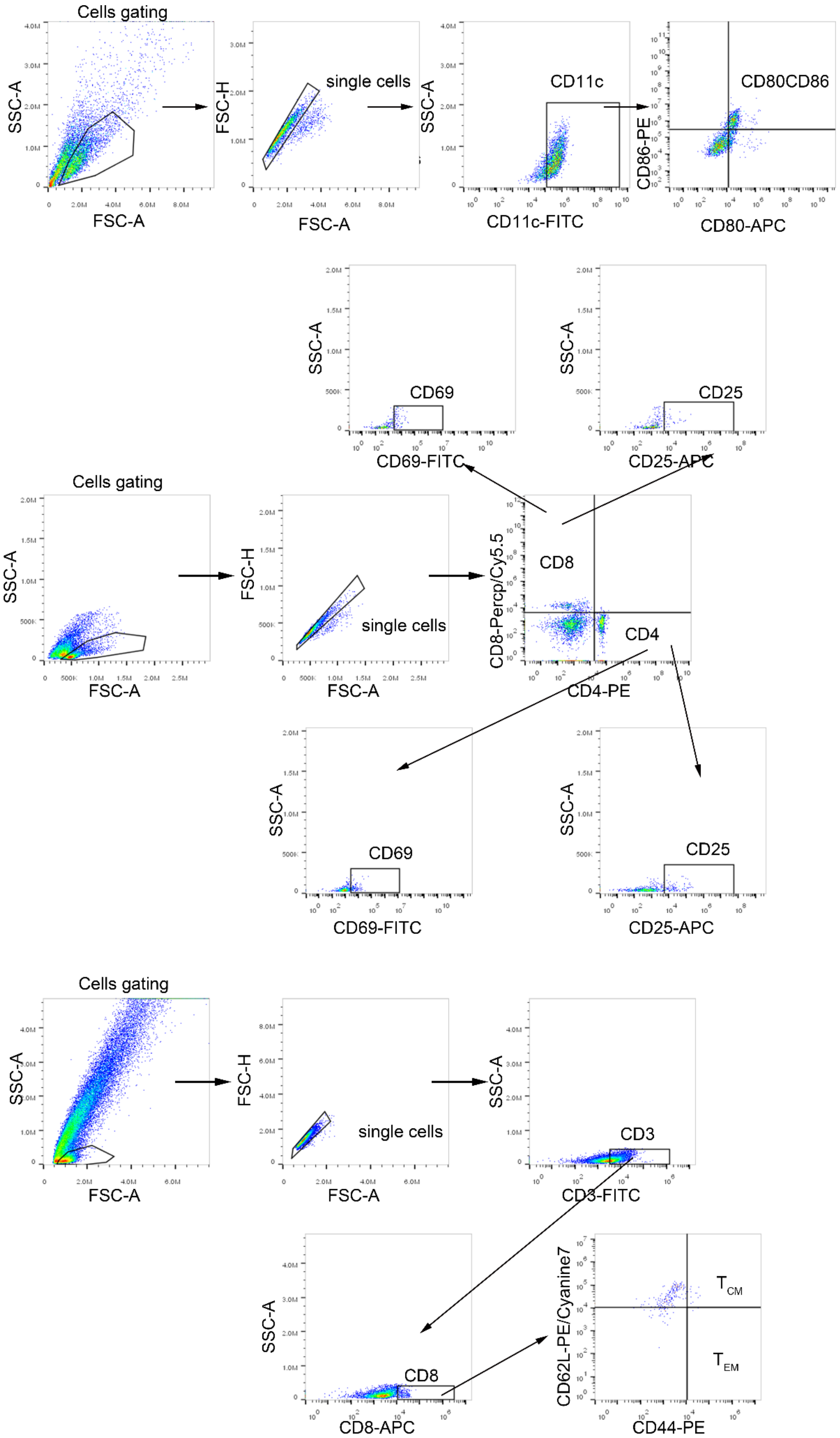

Supplementary Figure 19. Gating strategy for Figure 2 and Supplementary Figure 5.

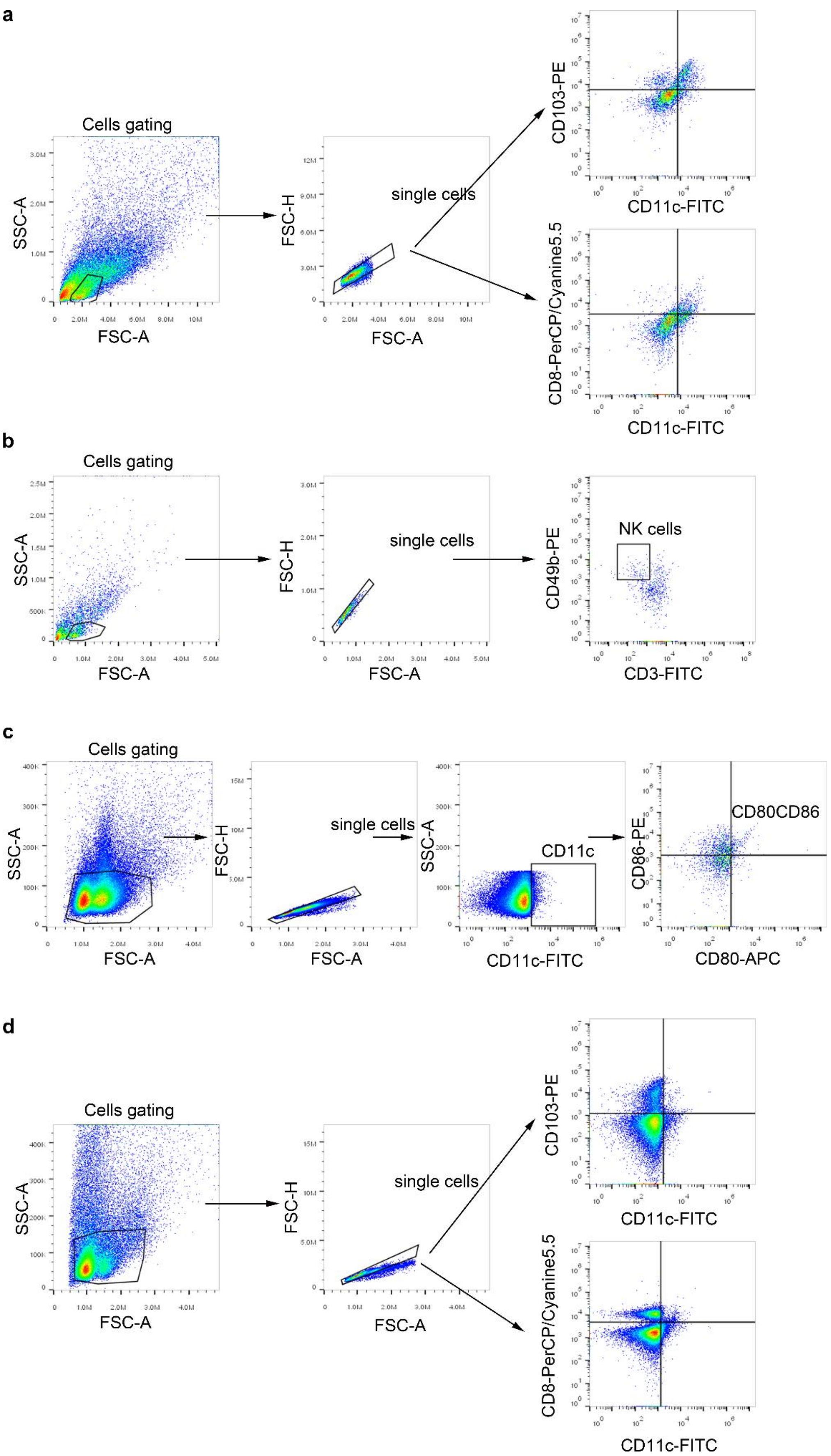

Supplementary Figure 20. Gating strategy for Figure 5. a, Gating strategy used for Figure

5d-e. **b**, Gating strategy used for Figure 5f. **c**, Gating strategy used for Figure 5g. **d**, Gating strategy used for Figure 5h-i.

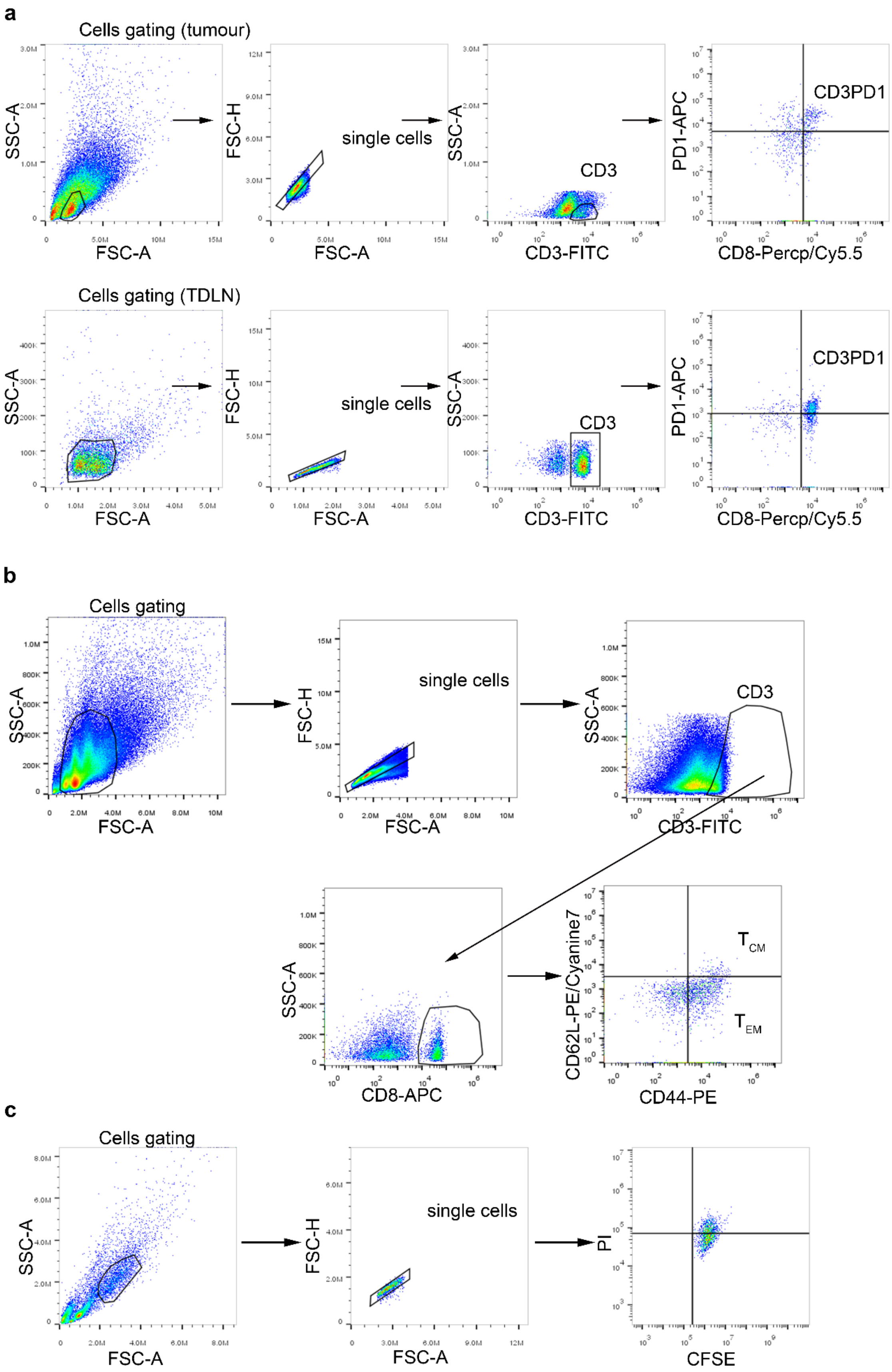

Supplementary Figure 21. Gating strategy for Figure 7. a, Gating strategy used for Figure 7a. **b**, Gating strategy used for Figure 7d-f. **c**, Gating strategy used for Figure 7g.

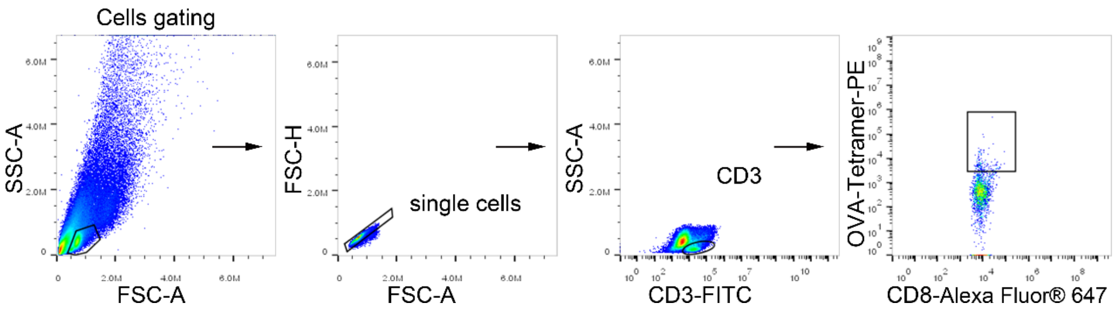

Supplementary Figure 22. Gating strategy for Figure 8e**.**

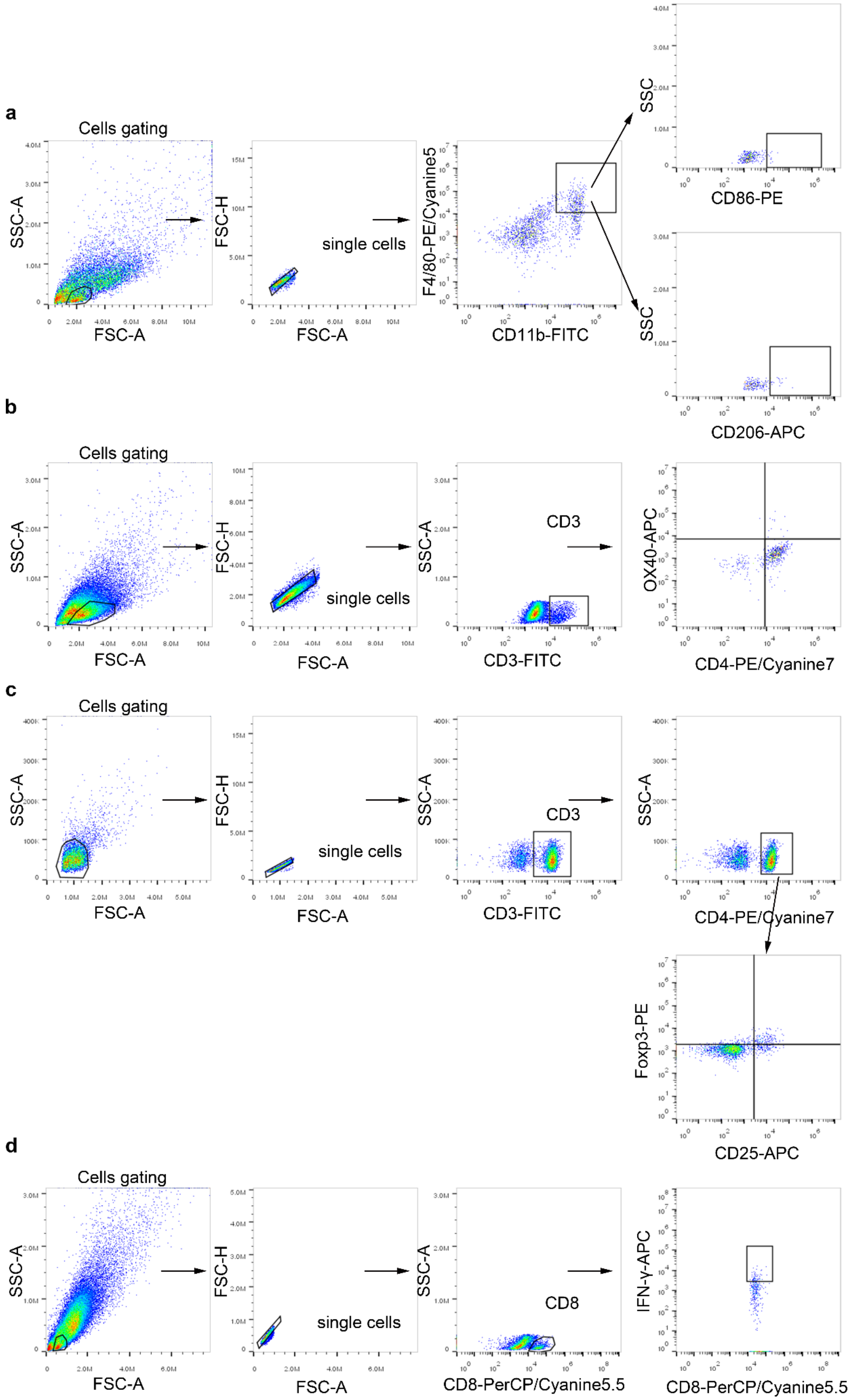

a
Cells gating
SSC-A
FSC-A
FSC-H
FSC-A
single cells
F4/80-PE/Cyanine5
CD11b-FITC
SSC
CD86-PE
SSC
CD206-APC
b
Cells gating
SSC-A
FSC-A
FSC-H
single cells
FSC-A
SSC-A
CD3
CD3-FITC
OX40-APC
CD4-PE/Cyanine7
c
Cells gating
SSC-A
FSC-A
FSC-H
single cells
FSC-A
SSC-A
CD3
CD3-FITC
SSC-A
CD4-PE/Cyanine7
Foxp3-PE
CD25-APC
d
Cells gating
SSC-A
FSC-A
FSC-H
single cells
FSC-A
SSC-A
CD8
CD8-PerCP/Cyanine5.5
IFN-γ-APC
CD8-PerCP/Cyanine5.5

Supplementary Figure 23. Gating strategy for Supplementary Figure 12. a, Gating strategy used for Supplementary Figure 12a-b. **b**, Gating strategy used for Supplementary Figure 12f. **c**, Gating strategy used for Supplementary Figure 12h. **d**, Gating strategy used for Supplementary Figure 12k.
