# Supplementary material for: Engineered Lactococcus lactis secreting Flt3L and OX40 ligand for in situ vaccination-based cancer immunotherapy
Source: Nat Commun. 2022 Dec 3;13:7466. doi: 10.1038/s41467-022-35130-7 (PMC9719518; doi:10.1038/s41467-022-35130-7)
Supplement: Supplementary file 3 — Description of Additional Supplementary Files [file 41467_2022_35130_MOESM3_ESM.pdf]

Description of Additional Supplementary Files

Supplementary Data 1: Peptide Identification List

Supplementary Data 2: Proteins Identification List
