# Supplementary material for: Engineered Lactococcus lactis secreting Flt3L and OX40 ligand for in situ vaccination-based cancer immunotherapy
Source: Nat Commun. 2022 Dec 3;13:7466. doi: 10.1038/s41467-022-35130-7 (PMC9719518; doi:10.1038/s41467-022-35130-7)
Supplement: Supplementary file 6 — Reporting Summary [file 41467_2022_35130_MOESM6_ESM.pdf]

nature portfolio

Corresponding author(s): Baorui Liu

Last updated by author(s): Nov 10, 2022

Reporting Summary

Nature Portfolio wishes to improve the reproducibility of the work that we publish. This form provides structure for consistency and transparency in reporting. For further information on Nature Portfolio policies, see our Editorial Policies and the Editorial Policy Checklist.

Statistics

For all statistical analyses, confirm that the following items are present in the figure legend, table legend, main text, or Methods section.

n/a	Confirmed	
☐	☒	The exact sample size (*n*) for each experimental group/condition, given as a discrete number and unit of measurement
☐	☒	A statement on whether measurements were taken from distinct samples or whether the same sample was measured repeatedly
☐	☒	The statistical test(s) used AND whether they are one- or two-sided<br>*Only common tests should be described solely by name; describe more complex techniques in the Methods section.*
☐	☒	A description of all covariates tested
☐	☒	A description of any assumptions or corrections, such as tests of normality and adjustment for multiple comparisons
☐	☒	A full description of the statistical parameters including central tendency (e.g. means) or other basic estimates (e.g. regression coefficient) AND variation (e.g. standard deviation) or associated estimates of uncertainty (e.g. confidence intervals)
☐	☒	For null hypothesis testing, the test statistic (e.g. *F*, *t*, *r*) with confidence intervals, effect sizes, degrees of freedom and *P* value noted<br>*Give P values as exact values whenever suitable.*
☒	☐	For Bayesian analysis, information on the choice of priors and Markov chain Monte Carlo settings
☒	☐	For hierarchical and complex designs, identification of the appropriate level for tests and full reporting of outcomes
☒	☐	Estimates of effect sizes (e.g. Cohen's *d*, Pearson's *r*), indicating how they were calculated

Our web collection on statistics for biologists contains articles on many of the points above.

Software and code

Policy information about availability of computer code

Data collection	BD Accuri C6 (BD Bioscience, USA) was used for flow-cytometric data collection. Images of the cells were collected on a confocal laser scanning microscopy (Leica, Germany). The sizes of subcutaneous tumors were measured with digital calipers. ELISA results were recorded with SpectraMax® i3x multi-mode microplate reader (Molecular Devices) using SoftMax® Pro 6 software (version 6.4.2). The releasing sfGFP protein was detected using a Varioskan Lux microplate reader (Thermo Fisher Scientific). CRi Maestro In Vivo Imaging System (Cambridge Research & Instrumentation, Massachusetts, USA) was used to analyze near-infrared imaging.
Data analysis	FlowJo v10 was used for the analysis of FACS data. GraphPad Prism 8 was used for plotting and statistical analysis. Image-Pro Plus 6.0 was adopted to analyze immunohistochemistry analysis. Quantification of gene expression and differential expression analysis was performed using DESeq2 Bioconductor package. GO network analysis of significantly upregulated genes in tumours were analyzed by Cytoscape software.

For manuscripts utilizing custom algorithms or software that are central to the research but not yet described in published literature, software must be made available to editors and reviewers. We strongly encourage code deposition in a community repository (e.g. GitHub). See the Nature Portfolio guidelines for submitting code & software for further information.

Data

Policy information about availability of data

All manuscripts must include a data availability statement. This statement should provide the following information, where applicable:

- Accession codes, unique identifiers, or web links for publicly available datasets
- A description of any restrictions on data availability
- For clinical datasets or third party data, please ensure that the statement adheres to our policy

The main data supporting the results in this study are available within the paper and its Supplementary Information. Source data are provided with this paper. The

RNA-seq data generated in this study have been deposited in the GSA database under accession code CRA008825 (https://ngdc.cncb.ac.cn/gsa/browse/CRA008825). Functionally related GO terms for biological processes were analyzed by GOSeq (v1.34.1), while Kyoto Encyclopedia of Genes and Genomes (KEGG) enrichment analysis used the database (http://en.wikip edia.org/wiki/KEGG).

Field-specific reporting

Please select the one below that is the best fit for your research. If you are not sure, read the appropriate sections before making your selection.

[x] Life sciences [ ] Behavioural & social sciences [ ] Ecological, evolutionary & environmental sciences

For a reference copy of the document with all sections, see nature.com/documents/nr-reporting-summary-flat.pdf

Life sciences study design

All studies must disclose on these points even when the disclosure is negative.

Sample size	Sample size was determined based on previous publications containing similar procedure to maintain the balance between reaching the statistical significance and minimizing the number of animal/reagents use. Published papers was used as references (PMID: 35013574; 34233949; 31185213; 35058589)
Data exclusions	No data were excluded from the analyses.
Replication	All experiments and in vitro assays were repeated in at least three independent experiments. RNA-seq was from one experiment and key discoveries were validated with other assays.
Randomization	The samples were randomly grouped.
Blinding	No blinding. Blinding was not needed in the study because conditions were well controlled with syngenic mice. Blinding is not typically used in the field.

Reporting for specific materials, systems and methods

We require information from authors about some types of materials, experimental systems and methods used in many studies. Here, indicate whether each material, system or method listed is relevant to your study. If you are not sure if a list item applies to your research, read the appropriate section before selecting a response.

Materials & experimental systems

n/a	Involved in the study	
[ ]	[x]	Antibodies
[ ]	[x]	Eukaryotic cell lines
[x]	[ ]	Palaeontology and archaeology
[ ]	[x]	Animals and other organisms
[x]	[ ]	Human research participants
[x]	[ ]	Clinical data
[x]	[ ]	Dual use research of concern

Methods

n/a	Involved in the study	
[x]	[ ]	ChIP-seq
[ ]	[x]	Flow cytometry
[x]	[ ]	MRI-based neuroimaging

Antibodies

Antibodies used

Flow cytometry: Antibodies to CD11c (N418, FITC, 117306), CD11c (N418, PE, 117308), CD80 (16-10A1, APC, 104714), CD86 (GL-1, PE, 105008), CD8a (53-6.7, PerCP/Cyanine5.5, 100734), CD103 (2E7, PE, 121406), CD3 (500A2, FITC, 152304), PD1 (29F.1A12, APC, 135210), CD4 (GK1.5, PE, 100408), CD8a (53-6.7, APC, 100712), CD25 (PC61, FITC, 102006), CD69 (H1.2F3, PE/Cyanine7, 104512), CD4 (GK1.5, PE/Cyanine7, 100422), CD25 (PC61, APC, 102012), FoxP3 (MF-14, PE, 126404), CD44 (IM7, PE, 103008), CD62L (MEL-14, PE/Cyanine7, 104418), CD11b (M1/70, APC, 101212), F4/80 (BM8, PE/Cyanine5, 123112), CD206 (C068C2, PE, 141706), CD134 (OX-86, APC, 119414), CD49b (DX5, PE, 108908), CD135 (A2F10, APC, 135310), IFN-γ (XMG1.2, APC, 505810) were purchased from Biolegend. Anti-CD8 (Mouse) mAb-Alexa Fluor® 647 (KT15) was obtained from MBL. All antibodies were diluted 1:100. H-2Kb OVA Tetramer-SIINFEKL (TS-5001-1C) was purchased from MBL and performed according to the instructions. Intracellular staining for FoxP3 was performed using the True-Nuclear™ Transcription Factor Buffer Set (Biolegend).

For histology: CD8 (EPR21769, Abcam) and PD-1 (EPR20665, Abcam) were purchased from Abcam company.

For treatments: anti-PD1 (clone 29F.1A12, BioXCell) were used at 100 ug per i.p. dose. Depletions of immune cells were done using antibodies against anti-mouse CD8 (clone 2.43, BioXCell, 400 µg per injection twice weekly), anti-mouse CD4 (GK1.5, BioXCell, 200 µg per injection weekly), anti-mouse CSF1R (CD115, BioXCell, 300 µg per injection every other day) and anti-ASGM1 (anti-mouse Asialo-GM1, 50 µl per injection twice weekly) .

For western blot, anti-Flt3L antibody (Abcam ab231192) and anti-His antibody (Cell Signaling Technology #12698S) were used to analyze peotein expressed by the engineered bacteria. Proteins of stimulated BMDCs were confirmed by WB analysis using an anti-TLR1 antibody (Abcam, ab37068), anti-TLR2 antibody (Abcam, ab209216), anti-TLR6 antibody (Cell Signaling Technology #12717), β-

actin antibody (Cell Signaling Technology #4967), anti-Phospho-NF-κB p65 (Cell Signaling Technology #3033S) and anti-Histone H3 (Cell SignalingTechnology #4499S). All antibodies were diluted 1:1000.

Validation

All antibodies are from commercial companies and are well validated by manufacturer and widely used in researches. Their validation data are available on the manufacturer's websites.
CD11c (N418, FITC, 117306) https://www.biolegend.com/en-us/products/fitc-anti-mouse-cd11c-antibody-1815

CD11c (N418, PE, 117308) https://www.biolegend.com/en-us/products/pe-anti-mouse-cd11c-antibody-1816

CD80 (16-10A1, APC, 104714) https://www.biolegend.com/en-us/products/apc-anti-mouse-cd80-antibody-2340

CD86 (GL-1, PE, 105008) https://www.biolegend.com/en-us/products/pe-anti-mouse-cd86-antibody-256

CD8a (53-6.7, PerCP/Cyanine5.5, 100734) https://www.biolegend.com/en-us/products/percp-cyanine5-5-anti-mouse-cd8a-antibody-4255

CD103 (2E7, PE, 121406) https://www.biolegend.com/en-us/products/pe-anti-mouse-cd103-antibody-3574

CD3 (500A2, FITC, 152304) https://www.biolegend.com/en-us/products/fitc-anti-mouse-cd3epsilon-antibody-13685

PD1 (29F.1A12, APC, 135210) https://www.biolegend.com/en-us/products/apc-anti-mouse-cd279-pd-1-antibody-6497

CD4 (GK1.5, PE, 100408) https://www.biolegend.com/en-us/products/pe-anti-mouse-cd4-antibody-250

CD8a (53-6.7, APC, 100712) https://www.biolegend.com/en-us/products/apc-anti-mouse-cd8a-antibody-150

CD25 (PC61, FITC, 102006) https://www.biolegend.com/en-us/products/fitc-anti-mouse-cd25-antibody-422

CD69 (H1.2F3, PE/Cyanine7, 104512) https://www.biolegend.com/en-us/products/pe-cyanine7-anti-mouse-cd69-antibody-3168

CD4 (GK1.5, PE/Cyanine7, 100422) https://www.biolegend.com/en-us/products/pe-cyanine7-anti-mouse-cd4-antibody-1919

CD25 (PC61, APC, 102012) https://www.biolegend.com/en-us/products/apc-anti-mouse-cd25-antibody-420

FoxP3 (MF-14, PE, 126404) https://www.biolegend.com/en-us/products/pe-anti-mouse-foxp3-antibody-4660

CD44 (IM7, PE, 103008) https://www.biolegend.com/en-us/products/pe-anti-mouse-human-cd44-antibody-2206

CD62L (MEL-14, PE/Cyanine7, 104418) https://www.biolegend.com/en-us/products/pe-cyanine7-anti-mouse-cd62l-antibody-1922

CD11b (M1/70, APC, 101212) https://www.biolegend.com/en-us/products/apc-anti-mouse-human-cd11b-antibody-345

F4/80 (BM8, PE/Cyanine5, 123112) https://www.biolegend.com/en-us/products/pe-cyanine5-anti-mouse-f4-80-antibody-4069

CD206 (C068C2, PE, 141706) https://www.biolegend.com/en-us/products/pe-anti-mouse-cd206-mmr-antibody-7424

CD134 (OX-86, APC, 119414) https://www.biolegend.com/en-us/products/apc-anti-mouse-cd134-ox-40-antibody-8305

CD49b (DX5, PE, 108908) https://www.biolegend.com/en-us/products/pe-anti-mouse-cd49b-pan-nk-cells-antibody-234

CD135 (A2F10, APC, 135310) https://www.biolegend.com/en-us/products/apc-anti-mouse-cd135-antibody-6284

IFN-γ (XMG1.2, APC, 505810) https://www.biolegend.com/en-us/products/apc-anti-mouse-ifn-gamma-antibody-993

Anti-CD8 (Mouse) mAb-Alexa Fluor® 647 (KT15) http://www.mbl-chinawide.cn/search012?keyword=anti-CD8

H-2Kb OVA Tetramer-SIINFEKL (TS-5001-1C) http://www.mbl-chinawide.cn/search012?keyword=TS-5001-1C

CD8 (EPR21769, Abcam) https://www.abcam.cn/cd8-alpha-antibody-epr21769-ab217344.html

PD-1 (EPR20665, Abcam) https://www.abcam.cn/pd1-antibody-epr20665-ab214421.html

anti-PD1 (clone 29F.1A12, BioXCell) https://bxcell.com/product/invivoplus-anti-mouse-pd-1-cd279/

anti-mouse CD8 (clone 2.43, BioXCell, 400 μg per injection twice weekly) https://bxcell.com/product/invivoplus-anti-m-lyt-2-2-cd8a/

anti-mouse CD4 (GK1.5, BioXCell, 200 μg per injection weekly) https://bxcell.com/product/invivoplus-anti-m-cd4/

anti-mouse CSF1R (CD115, BioXCell, 300 μg per injection every other day) https://bxcell.com/product/invivoplus-anti-mouse-csf1r-cd115/

anti-ASGM1 (anti-mouse Asialo-GM1, 50 μl per injection twice weekly) Asialo GM1 Polyclonal Antibody, Functional Grade (16-6507-39) (thermofisher.cn)

anti-Flt3L antibody (Abcam ab231192) https://www.abcam.cn/flt3-ligandflt3l-antibody-ab231192.html

anti-His antibody (Cell Signaling Technology #12698S) https://www.cellsignal.cn/products/primary-antibodies/his-tag-d3i1o-xp-rabbit-mab/12698?site-search-type=Products&N=4294956287&Ntt=%2312698s&fromPage=plp&_requestid=8743203

anti-TLR1 antibody (Abcam, ab37068) https://www.abcam.cn/tiltlr1-antibody-ab37068.html

anti-TLR2 antibody (Abcam, ab209216) https://www.abcam.cn/tlr2-antibody-epr20302-119-ab209216.html

anti-TLR6 antibody (Cell Signaling Technology #12717) https://www.cellsignal.com/products/primary-antibodies/toll-like-receptor-6-d1z8b-rabbit-mab/12717?site-search-type=Products&N=4294956287&Ntt=tlr6&fromPage=plp

β-actin antibody (Cell Signaling Technology #4967) https://www.cellsignal.cn/products/primary-antibodies/b-actin-antibody/4967?site-search-type=Products&N=4294956287&Ntt=%234967%29&fromPage=plp&_requestid=8743422

anti-Phospho-NF-κB p65 (Cell Signaling Technology #3033S) https://www.cellsignal.cn/products/primary-antibodies/phospho-nf-kb-p65-ser536-93h1-rabbit-mab/3033?site-search-type=Products&N=4294956287&Ntt=%233033s&fromPage=plp&_requestid=8743573

anti-Histone H3 (Cell Signaling Technology #4499S) https://www.cellsignal.cn/products/primary-antibodies/histone-h3-d1h2-xp-rabbit-mab/4499?site-search-type=Products&N=4294956287&Ntt=%234499s&fromPage=plp&_requestid=8743728

Eukaryotic cell lines

Policy information about cell lines

Cell line source(s)	Cell lines B16F10, 4T1 and CT26 cells were obtained from the Cell Bank of Shanghai Institute of Biochemistry and Cell Biology, and cultured following vendor instructions. 4T1-GFP-Luc cells were generated by transfection of the 4T1 cell line with pGreenFire lentiviral vector (System Biosciences). CT26-GFP-Luc cells were generated by transfection of the CT26 cell line with pGreenFire lentiviral vector (System Biosciences). MC38-GFP-Luc cells were generated by transfection of the MC38 cell line with pGreenFire lentiviral vector (System Biosciences). B16F10-OVA cell line was generated by lentivirus-based infection of a vector encoding OVA protein in B16F10 tumor cells.
Authentication	Each cell line was maintained separately and stocked in early passages, to minimize contamination and to preserve cell identity. No further authentication was performed before use.
Mycoplasma contamination	Our laboratory peformed mycoplasma testing on all cells cultured every month and all cell lines used tested negative for mycoplasma contamination.
Commonly misidentified lines (See ICLAC register)	No misidentified cell lines listed by ICLAC were used.

Animals and other organisms

Policy information about studies involving animals; ARRIVE guidelines recommended for reporting animal research

Laboratory animals	BALB/c male/female mice and C57BL/6 male mice aged 5-6 weeks were purchased from Shanghai Sippr-BK laboratory animal Co. Ltd. (Shanghai, China) and kept in the specific pathogen-free (SPF) Laboratory Animal Center of Affiliated Nanjing Drum Tower Hospital of

	Nanjing University Medical School.
Wild animals	The study did not involve wild animals.
Field-collected samples	The study did not involve any samples collected from field.
Ethics oversight	All animal experimental protocols were approved by the Laboratory Animal Care and Use Committee of the Affiliated Nanjing Drum Tower Hospital of Nanjing University Medical School.

Note that full information on the approval of the study protocol must also be provided in the manuscript.

Flow Cytometry

Plots

Confirm that:

☒ The axis labels state the marker and fluorochrome used (e.g. CD4-FITC).

☒ The axis scales are clearly visible. Include numbers along axes only for bottom left plot of group (a 'group' is an analysis of identical markers).

☒ All plots are contour plots with outliers or pseudocolor plots.

☒ A numerical value for number of cells or percentage (with statistics) is provided.

Methodology

Sample preparation	Tumor tissues, TDLNs and spleens were taken out from mice. Single cell suspension from the spleen and TDLNs was prepared by mechanical trituration method while tumor tissues minced into small pieces were digested with collagenase type IV (1 mg/mL, Sigma) for 2 h at at room temperature with gentle agitation. All samples were then resuspended in ice-cold NS, stained with specific antibodies for 20 min in at room temperature in darks, and then washed before analysis.
Instrument	Cells were analysed using BD Accuri C6 (BD Bioscience, USA) .
Software	FlowJo v10.
Cell population abundance	For in vitro analysis, at least 2000 live CD11c DCs were collected for analysis. For in vivo analysis, at least 20000 live lymphocytes were collected for analysis.
Gating strategy	Described in the relevant figures.

☒ Tick this box to confirm that a figure exemplifying the gating strategy is provided in the Supplementary Information.
